# Biocompatible Gas Plasma Treatment Affects Secretion Profiles but Not Osteogenic Differentiation in Patient-Derived Mesenchymal Stromal Cells

**DOI:** 10.3390/ijms23042038

**Published:** 2022-02-12

**Authors:** Maximilian Fischer, Janosch Schoon, Eric Freund, Lea Miebach, Klaus-Dieter Weltmann, Sander Bekeschus, Georgi I. Wassilew

**Affiliations:** 1 Center for Orthopaedics, Trauma Surgery and Rehabilitation Medicine, University Medicine Greifswald, 17475 Greifswald, Germany; maximilian.fischer@med.uni-greifswald.de (M.F.); georgi.wassilew@med.uni-greifswald.de (G.I.W.); 2 ZIK *plasmatis*, Leibniz Institute for Plasma Science and Technology (INP), Felix-Hausdorff-Str. 2, 17489 Greifswald, Germany; eric.freund@inp-greifswald.de (E.F.); lea.miebach@inp-greifswald.de (L.M.); weltmann@inp-greifswald.de (K.-D.W.); 3 Department of General, Visceral, Thorax, and Vascular Surgery, Greifswald University Medical Center, 17475 Greifswald, Germany

**Keywords:** mesenchymal stromal cells, cold atmospheric pressure plasma, arthroplasty, cold physical plasma, plasma medicine, osteogenic differentiation, reactive oxygen species

## Abstract

Cold physical plasma (CPP), a partially ionized gas that simultaneously generates reactive oxygen and nitrogen species, is suggested to provide advantages in regenerative medicine. Intraoperative CPP therapy targeting pathologies related to diminished bone quality could be promising in orthopedic surgery. Assessment of a clinically approved plasma jet regarding cellular effects on primary bone marrow mesenchymal stromal cells (hBM-MSCs) from relevant arthroplasty patient cohorts is needed to establish CPP-based therapeutic approaches for bone regeneration. Thus, the aim of this study was to derive biocompatible doses of CPP and subsequent evaluation of human primary hBM-MSCs’ osteogenic and immunomodulatory potential. Metabolic activity and cell proliferation were affected in a treatment-time-dependent manner. Morphometric high content imaging analyses revealed a decline in mitochondria and nuclei content and increased cytoskeletal compactness following CPP exposure. Employing a nontoxic exposure regime, investigation on osteogenic differentiation did not enhance osteogenic capacity of hBM-MSCs. Multiplex analysis of major hBM-MSC cytokines, chemokines and growth factors revealed an anti-inflammatory, promatrix-assembling and osteoclast-regulating secretion profile following CPP treatment and osteogenic stimulus. This study can be noted as the first in vitro study addressing the influence of CPP on hBM-MSCs from individual donors of an arthroplasty clientele.

## 1. Introduction

Cold physical plasma (CPP), generated by adding energy to a neutral gas, contains a plethora of reactive oxygen and nitric species (ROS/RNS) and is employed to combat defective wound healing [1]. In recent years, novel biomedical applications were suggested to take advantage of such gas plasma technology by bridging physical engineering and medical advances in, for instance, regenerative medicine [2]. One important application of CPP is the treatment of implant surfaces to enhance biocompatibility and ingrowth capacity [3]. Many commonly used titanium-based implant components in arthroplasty are vacuum plasma sprayed to augment an open-pore layer resulting in increased bone integration [4]. Moreover, material studies in dentistry provided beneficial effects of CPP-induced surface modifications. Argon-plasma treatment of titanium and zirconium surfaces enhanced cell adhesion and viability of the MC3T3-E1 osteoblastic cell line in vitro [5,6]. The primary biomedical response of CPP on mammalian tissue or cells is based on changes in the liquid environment around cells through the production of ROS/RNS, organic radicals and electrons and energetic ions, placing the field in the heart of redox biology and medicine [2,7]. The argon-driven kINPen is the first CPP jet worldwide approved as medical device [8]. The use of argon as preassembled working gas makes the reactive species composition easy controllable and enables standardized medical applications. Due to its various application options, the kINPen is under investigation in many different fields of medicine. In preclinical research, CPP at high doses induced apoptosis in cancer cells and opened the door for novel antitumor therapies [8,9]. In vitro studies showed enhanced proliferation of keratinocytes and secretion of growth factors under CPP treatment with the kINPen at biocompatible dosing [10]. In dermatology, the kINPen is becoming standard medical care for treating chronic wounds and pathogen-based skin disease [1]. kINPen treatment shows numerous promising properties, including infection control [11] and hemostasis promotion [12], which might also be utilized in intraoperative approaches. Moreover, studies have demonstrated effective antibacterial effects of plasma treatment using the kINPen, including an effect on multidrug-resistant pathogens [13,14].

Infection, trauma, and degeneration of musculoskeletal tissues are associated with acute or chronic inflammation and tissue loss [15]. Thus, anti-inflammatory and tissue regenerative CPP at biocompatible doses might be a promising treatment option for improving future medical care in orthopedic and trauma surgery. Particularly concerning the healing of bone defects after injury or orthopedic surgery, osteoblasts are crucial for bone mineralization in the bone healing cascade and retrieval of vital bone remodeling [16]. Studies on MC3T3-E1 osteoblast cell lines showed promising results on alkaline phosphatase (ALP) activity after short-term helium-driven CPP treatment. Enhanced ALP activity after CPP treatment for 5 s over 5 consecutive days and vital cell proliferation indicated CPP feasibility at biocompatible doses [17]. Sufficient osseointegration of implant components is driven by multipotent adult mesenchymal stromal cells (MSCs) with the ability to differentiate into osteoblasts, chondrocytes and adipocytes [18]. Due to their self-renewal capacity, multipotentiality, and straightforward isolation from different tissues, MSCs are a promising cell source for intravascular anti-inflammatory therapy, tissue engineering and local cell therapeutic approaches in musculoskeletal regeneration [19]. Especially human bone-marrow-derived MSCs (hBM-MSCs) are of particular importance for therapies addressing bone regeneration, because of their accessibility during standard operations in orthopedic surgery, e.g., arthroplasty, and their high affinity to differentiate into the osteoblastic lineage [20]. Yet, knowledge about the effects of CPP on hBM-MSCs’ functionality is limited to reports about periodontal and adipose-derived MSCs [21,22]. In vitro treatment of stromal cells from adipose tissue with a helium-driven CPP device showed a slightly higher proliferation rate and no DNA-damaging effects in comparison to control conditions [21]. In contrast, proliferation, migration, and adhesion of periodontal ligament MSCs were diminished by gas plasma treatment, and cellular ALP activity was increased, suggesting osteoinductive effects in vitro [22]. However, the implications of these few studies are insufficient to evaluate the potential of CPP in orthopedics. Thus, there is a need to investigate the application of clinically relevant argon-derived CPP on primary hBM-MSCs from patients undergoing primary hip arthroplasty.

This study investigated hBM-MSCs from a clinically relevant patient cohort after in vitro exposure to CPP using the clinically approved kINPen argon jet. The aims of this study were (1) to quantify ROS/RNS levels and ROS/RNS persistency in cell culture media, (2) to derive biocompatible treatment doses from in vitro evaluation of cell viability, proliferation and morphology and (3) to elucidate the osteogenic and immunomodulatory profiles of hBM-MSCs exposed to biocompatible (nontoxic) CPP treatment times. We hypothesized that CPP improves osteogenic differentiation of hBM-MSCs at biocompatible doses. Hence, as an osteoinductive and anti-inflammatory approach targeting pathologies related to diminished peri-implant bone quality, intraoperative CPP therapy could be promising in orthopedic surgery. We follow the overall aim to introduce CPP as a potential future medical treatment of human musculoskeletal cells and tissues to foster endogenous regeneration.

## 2. Results

### 2.1. Quantification of Reactive Species in Cell Culture Media

**Figure 1:**
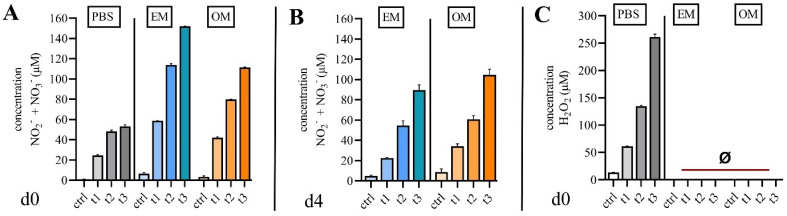
CPP generates reactive species in cell culture media. (**A**) Quantification of nitrate and nitrite immediately after treatment with CPP reveals a dose-dependent increase of nitrogen species in PBS (control), expansion media (EM) and osteogenic media (OM). (**B**) Quantification of nitrate and nitrite four days after treatment with CPP reveals persistency of nitrogen species which is more pronounced in OM than in EM. (**C**) Cytotoxic hydrogen peroxide was not generated in EM and OM. (mean ± SD, *n* = 3).

Gas plasmas are known to generate short-lived ROS/RNS, which react to stable species in liquids. Biological effects of CPP treatment are associated with the antioxidative capacity of the liquids. Thus, NO_2_^-^ and NO_3_^-^ as well as H_2_O_2_ were quantified in PBS, expansion medium (EM) and osteogenic medium (OM) following CCP exposure. Exposure times were 30 s (t_1_), 60 s (t_2_) or 120 s (t_3_). NO_2_^-^ and NO_3_^-^ were detected in media immediately after CPP treatment (Figure 1A) and 96 h after CPP treatment (day four) (Figure 1B). Concentrations of NO_2_^-^ and NO_3_^-^ were higher in EM, whereas the persistency of NO_2_^-^ and NO_3_^-^ was more pronounced in OM. H_2_O_2_ was detected in PBS in a treatment-time-dependent manner but was not detected in EM and OM (Figure 1C).

### 2.2. Characterization of Mesenchymal Stromal Cells

**Figure 2:**
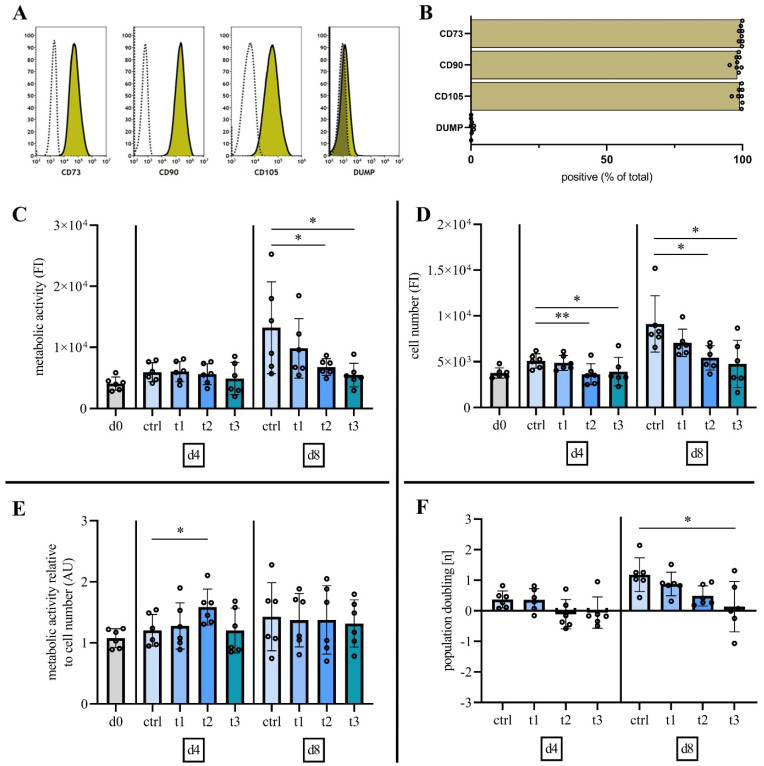
Surface marker expression analysis indicates MSC specific profiles and CPP treatment of hBM-MSCs shows a dose-dependent decrease in cell viability, cell number and population doubling. (**A**,**B**) Flow cytometry analyses of hBM-MSC at cell culture passage 3 revealed expression of positive cell surface markers CD73, CD90 and CD105 and the absence of expression of negative markers CD14, CD19, CD34, CD45, HLA-DR (DUMP). (**C**) Quantification of metabolic activity 8 days after treatment reveals a dose-dependent decrease in cell viability. (**D**) 60 s (t2) and 120 s (t3) of treatment with CPP reduces cell numbers at d4 and d8. (**E**) Normalization to cell number indicates an increase of hBM-MSCs metabolic activity 4 days after treatment with CPP for 60 s (t2). (**F**) Negative population doubling at day 4 after 60 s (t2) and 120 s (t3) of treatment with CPP and negative population doubling at day 8 after 90 s (t3) of treatment with CPP indicates cytotoxic effects. (mean ± SD, *n* = 6, one-way ANOVA with Bonferroni’s post hoc test, levels of significance: * *p* < 0.05, ** *p* < 0.01).

To evaluate hBM-MSC characteristics as defined by the ISCT criteria [23], the surface marker expression (CD73, CD90, CD105, CD14, CD19, CD34, CD45, HLA-DR) of hBM-MSC from all donors were analyzed at culture passage three. Flow cytometry analysis indicated successful isolation of hBM-MSCs by expression (mean ± SD) of positive hBM-MSC markers (CD73, 99.52% ± 0.55%; CD90, 98.13% ± 1.29%; CD105, 99.04% ± 1.34%) and marginal expression (DUMP, 0.37% ± 0.46%) of negative markers (DUMP) CD14, CD19, CD34, CD45, HLA-DR (Figure 2A,B).

### 2.3. Cold Physical Plasma Alters Cell Viability and Proliferation Capacity

Following quantification of reactive species for the defined treatment regime and characterization of hBM-MSCs, the question was whether different treatment times, i.e., different concentrations of reactive species alter cell viability and proliferation capacity of primary hBM-MSCs.

Resazurin-based viability assay revealed no alteration of metabolic activity at day four after CPP treatment at all doses (Figure 2C). Eight days after CPP treatment, cell viability was reduced in a dose-dependent manner. This reduction was statistically significant after CPP exposure for 60 s and 120 s (Figure 2C). A statistically significant reduction of cell number was detected on days 4 and 8 after treatment for 60 s and 120 s (Figure 2D). Normalization of cell viability to total cell number (DNA quantification) revealed a statistically significant increase of cell viability (metabolic activity) at day 4 after a 60 s treatment with CPP (Figure 2E). Normalization reveals that the reduced cell viability following treatment with CPP is related to significantly reduced cell number. To investigate the proliferation capacity after CPP treatment, the number of population doublings at days 4 and 8 were calculated (Figure 2F). Negative values were detected at day 4 after CPP treatment, which indicates cell number reduction due to cell death. On day 8, cell proliferation was significantly reduced after CPP treatment for 120 s.

Taken together, cell numbers were significantly reduced after a single treatment with CPP for 60 s and 120 s in a dose-dependent manner. However, no statistically significant effects were detected after treatment for 30 s.

### 2.4. Exposure to Cold Physical Plasma Affects hBM-MSC Morphology

**Figure 3:**
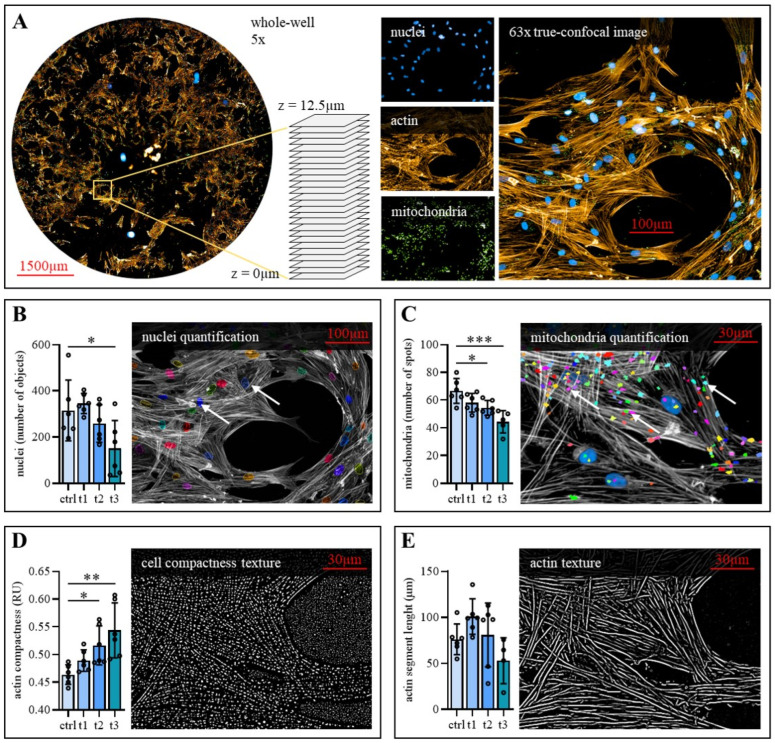
Dose-dependent morphology changes after CPP treatment of hBM-MSCs. (**A**) Representative high content images at 5× and 63× magnification and focus stacking (12.5 µm) indicate nuclei, actin and mitochondria staining. Automated imaging of whole-wells at 63× magnification indicates (**B**) a decreased nuclei number after 120 s (t3) CPP treatment, (**C**) decreased numbers of mitochondria after 60 s and 120 s CPP treatment, (**D**) an increased cell compactness after 60 s and 120 s CPP treatment and (**E**) no statistically significant changes in actin segment length after CPP treatment. (Mean ± SD, *n* = 6, one-way ANOVA with Bonferroni’s post hoc test, levels of significance: * *p* < 0.05, ** *p* < 0.01, *** *p* < 0.001).

MSCs are distinguished by a characteristic morphology and a complex actin cytoskeleton, which is crucial for cellular function. Therefore, morphological changes of representative hBM-MSCs from one donor were analyzed after CPP treatment (Figure S1). High content imaging (Figure 3A) showed alterations in nucleus number, mitochondria number, actin compactness and actin segment length if compared to untreated control cells. CPP treatment reduces the number of nuclei, confirming the finding of reduced cell and mitochondria number in a dose-dependent manner (Figure 3B,C). In addition, exposure to CPP altered actin filament morphology. While actin compactness was significantly increased in CPP treated hBM-MSCs (Figure 3D), CPP exposure caused no significant decrease of actin segment length (Figure 3E).

Taken together, the cell morphology was significantly altered after a single treatment with CPP for 60 s and 120 s in a dose-dependent manner. However, no statistically significant effects were detected after treatment for 30 s. Considering these findings and findings regarding cell viability and proliferation, the 30 s/cm^2^ treatment with CPP did not induce cytotoxic effects on hBM-MSCs. It was thus applied for the in vitro experiments concerning osteogenic differentiation and secreted signaling molecules.

### 2.5. Matrix Mineralization Was Not Affected by Exposure to Cold Physical Plasma

To investigate the effects of the biocompatible CPP dose of 30 s/cm^2^ on osteogenic differentiation, hBM-MSCs were treated at day 0 and day 4 in expansion media and osteogenic media.

**Figure 4:**
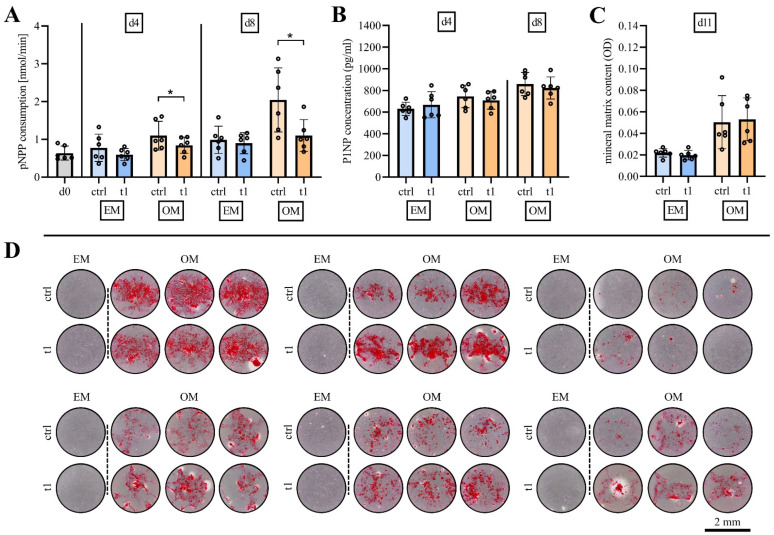
Biocompatible dose (t1, 30 s) of CPP does not increase osteogenic capacity of hBM-MSCs. (**A**) Alkaline phosphatase activity is reduced after treatment with CPP at day 8 of osteogenic differentiation (**B**) Quantification of P1NP in media supernatant indicates that synthesis of type I collagen is not affected following CPP treatment. (**C**) Quantification of mineralized matrix reveals that CPP treatment does not alter mineral matrix content at day 11 of osteogenic differentiation. (**D**) Representative phase contrast images of alizarin red-stained mineral matrix of MSCs from six bone marrow donors. (Mean ± SD, *n* = 6, paired samples t-test, level of significance: * *p* < 0.05).

The influence of CPP at early stages of osteogenic differentiation capacity was quantified at day 4 and day 8 of osteogenic differentiation. ALP activity was higher in hBM-MSCs cultured under osteogenic culture conditions (OM) in comparison to culture in expansion media (EM), indicating a successful experimental setup for osteogenic differentiation. CPP treatment causes a significant reduction of ALP activity of osteogenic induced hBM-MSCs compared to control at days 4 and 8 (Figure 4A). Matrix mineralization was quantified as another marker of osteogenic differentiation and pro-collagen type I (p1NP) was detected in cell culture supernatant. The p1NP concentration in cell culture supernatants showed an increase in osteogenic media at day 4 of osteogenic differentiation compared to cells cultured in expansion media. It was detected to be further increased in osteogenic media of hBM-MSCs after 8 days of osteogenic differentiation (Figure 4B). Treatment with CPP did not influence collagen I syntheses at both time points. Alizarin Red staining was used to quantify matrix mineralization at day 11 (Figure 4C). Quantitative analyses revealed no differences regarding matrix mineralization between CPP-treated and untreated hBM-MSCs. Stained matrix was documented by phase-contrast microscopy of every cell culture well (Figure 4D).

Taken together, CPP treatment at biocompatible dose did not show osteoinductive effects regarding collagen I synthesis, ALP activity and matrix mineralization.

### 2.6. Soluble Mediator Multiplex Analysis of Major MSC Secreted Signaling Molecules 

**Figure 5:**
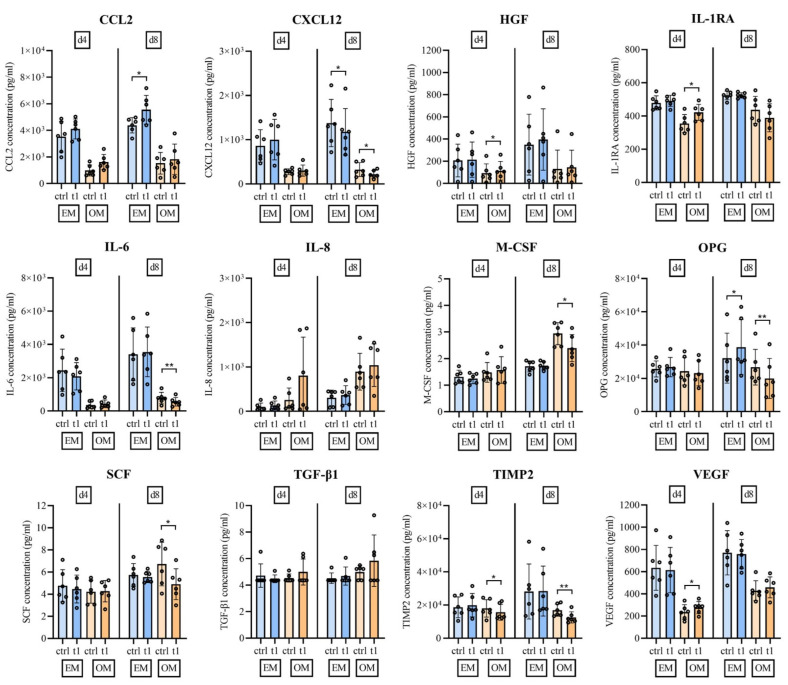
Multiplex analysis of cytokines, chemokines and growth factors in culture supernatant indicates regulation of factors following osteogenic differentiation and treatment with CPP. The biocompatible dose (t1, 30 s) of CPP leads to regulation of inflammation regulating factors growth factors, matrix and stem cell niche regulating factors and paracrine cell maintenance and function associated factors. (Mean ± SD, *n* = 6, data points are shown as the mean of a quadruplicate analysis of every sample, paired samples t-test, level of significance: * *p* < 0.05, ** *p* < 0.01).

Multiplex analysis was performed to evaluate whether CPP influences the release of important cytokines, chemokines and growth factors released by hBM-MSCs. To this end, 12 factors attributed to immunoregulatory function, matrix remodeling and bone metabolism of MSCs were selected (Figure 5).

The analysis showed that osteogenic stimulus strongly influenced the release of the analyzed signaling molecules. The mean concentrations of six analyzed factors were reduced/enhanced by a fold change of >2 at either day 4 or 8 of osteogenically differentiated hBM-MSCs compared to nondifferentiated hBM-MSCs. Fold decrease > 2: IL-6, CCL2, CXCL12, HGF, VEGF; fold increase > 2: IL-8 (Figure 5).

A paired-samples t-test was performed to analyze whether CPP-treated versus untreated hBM-MSCs leads to statistically significant changes regarding the release of cytokines, chemokines and growth factors. The release of wound healing associated CCL2 was increased at days 4 and 8 with and without osteogenic stimulus, which reached statistical significance at day 8 under nonosteogenic conditions. The MMPs inhibitor TIMP2 was significantly downregulated after CPP treatment at day 4 and day 8 of osteogenic differentiation. Concentrations of CXCL12 and SCF, two factors involved in hematopoietic stem-cell maintenance, were also significantly lower after CPP treatment at day 8 of osteogenic differentiation. Growth factors HGF and VEGF were both significantly upregulated following CPP treatment under osteogenic conditions at day 4. The secretion of anti-inflammatory IL-1RA was significantly upregulated by CPP treated cells at day 4 of osteogenic differentiation, and pro-inflammatory IL-6 was significantly downregulated at day 8 of osteogenic differentiation. Moreover, OPG, which captures RANKL to restrict osteoclast activity, was significantly upregulated at day 8 under nonosteogenic culture conditions and significantly downregulated at day 8 under osteogenic conditions. M-CSF, which is known to induce osteoclast differentiation was upregulated following CPP treatment and osteogenic stimulus at culture day 8 (Figure 5).

Taken together, treatment of hBM-MSCs with biocompatible CPP doses under osteogenic conditions leads to an anti-inflammatory, promatrix-assembling and osteoclast-regulating secretion profile.

## 3. Discussion

The study aimed to derive biocompatible doses of CPP for treating primary human BM-MSCs from various donors by in vitro evaluation of cell viability, proliferation capacity and morphological changes. Moreover, the study aimed to investigate the influence of CPP on cellular functionality, i.e., osteogenic differentiation and changes in cytokine and growth factor secretion, involved in immune cell recruitment, anti-inflammatory pathways, matrix remodeling and bone metabolism of hBM-MSCs isolated from a clinically relevant patient cohort. Treatment with CPP leads to a dose-dependent decrease of hBM-MSCs proliferation capacity, associated with morphological changes, indicating an exposure time of 30 s/cm^2^ to be a biocompatible dose for the treatment of hBM-MSCs. Using this treatment condition, effects on osteoinduction were minor, whereas CPP treatment leads to significant changes of hBM-MSCs secreted signaling molecules.

The study’s data show a dose-dependent decrease of cell viability and proliferation capacity after CPP-treatment for 60 s (t2) and 120 s (t3). In vitro CPP treatment is known to induce apoptosis in human cells in a dose-dependent manner [24]. In the present study a single treatment time of 30 s/cm^2^ is biocompatible for treating hBM-MSCs and does not influence cell viability and proliferation in vitro. This identified treatment time is in the mid-range (<16–50 s) of biocompatible CPP doses defined by other research groups [17,21,25,26,27]. Overall, in vitro CPP treatment regimes with experimental CPP are inconsistent and hardly comparable. The differences in the experimental setup, plasma devices, gas mixture and dosimetry (treatment time/cell number) could explain the divergences. The treatment of human adipose-tissue-derived MSCs and hBM-MSCs with helium-derived CPP was biocompatible at 50 s treatment time [21,25]. Tominami et al., used a comparable experimental setup but applied a treatment regime of 10 consecutive exposure periods, to define a biocompatible dose of 50 s for the osteoblastic precursor cell line MC3T3-E1 [17]. In the present study, an argon-plasma treatment of 30s was detected to be biocompatible, using a single-dose treatment regime that can be practical in routine. In the case of single CPP-treatment regimes of mesenchymal cell lines, biocompatible doses defined by other research groups were within the range of our data. Single nitrogen-plasma treatment of MC3T3-E1 osteoblasts was biocompatible at a treatment time <16 s [27], while argon-plasma treatment of L929 fibroblasts less than 20 s was defined as biocompatible [26].

The identified biocompatible CPP dose for the treatment of primary hBM-MSCs showed no enhancing effects on in vitro osteogenic differentiation, yet cellular ALP activity was found to be slightly reduced following CPP treatment. ALP is crucial for matrix mineralization and an early marker of osteogenic differentiation. In contrast to our findings, research on different osteoblastic cell lines revealed osteoinductive effects by upregulation of ALP and matrix mineralization after CPP-treatment [17,28,29]. These studies were conducted with immortalized murine or human cell lines. Thus, cell population and patient-specific variability cannot be depicted by these in vitro studies. In the present representative study cohort, the hBM-MSCs of only one patient showed increased matrix mineralization following CPP treatment, indicating that individual patient factors could play a role in response and nonresponse to CPP therapies. We are certain that preclinical testing of the clinically approved plasma source on primary human cells from various donors is necessary to obtain robust statements regarding the effect of a future plasma treatment in a clinical setup. 

Vital bone tissue homeostasis is characterized by a complex interplay of biomechanics, different matrix components and various cell types. Therefore, models that emulate this complexity should be chosen in the next step of preclinical validation of effects on vital bone. The application of microphysiological biomechanics and hypoxia should be considered to further approach in vitro bone models to the in vivo reality [30]. Recent achievements allow for perfused 3D culture of human bone with bone matrix mineralizing and absorbing cells, as well as the complex immune cell composition in the intertrabecular bone marrow [31]. In particular, the presence of immune cells may play a major role in inducing any effect of CPP on bone homeostasis. Anti-inflammatory effects induced by pro-proliferative and proapoptotic signaling cascades after plasma treatment of a monocyte cell line have been attributed to CPP [32]. Such effects may also positively impact bone homeostasis in inflammatory pathologies driven by enhanced macrophage differentiation and proliferation such as periprosthetic osteolysis [33]. Animal studies may also represent an opportunity to picture the complexity of bone tissue homeostasis and pathologies associated with disturbed bone remodeling. To our knowledge, there are no animal-based preclinical studies on the effects of CPP on bone. Conceivable approaches might be to study the effect of anti-inflammatory CPP on bone healing [34] and aseptic and septic osteolysis [35]. Another promising approach for CPP-related osteoinduction is the treatment of biomaterials with CPP. Surfaces of biomaterials can be oxidatively modified by CPP treatment. This modification (dependent on plasma conditions) can positively affect cell behavior in terms of osteogenic capacity [36,37,38].

Successful bone healing is conditioned by a complex multistep process of healing cascades including inflammatory, anti-inflammatory and proangiogenic cascades [39]. In particular, due to CPP’s angiogenic [40] and anti-inflammatory effects [41] in dermatology setups, testing CPP on bone healing is a promising approach. Besides the direct effects of CPP on immune cells, paracrine effects following CPP treatment should additionally be considered. BM-MSCs are attributed to a distinct immunomodulatory potential based on cytokine-dependent and cytokine-independent effects on, e.g., regulatory T cells [42]. It is conceivable that such effects can be influenced by pretreatment of BM-MSCs with CPP. In a recent in vitro study on the effects of a 60 s ambient air CPP treatment of a human osteoblast-like cell line, the upregulation of mainly pro-inflammatory cytokines (IL-1β, IL-6, IL-8, TNFα) was induced [43]. In contrast, no pro-inflammatory cytokine release was detected at biocompatible experimental conditions chosen in the presented study. Effects on anti-inflammatory (IL-1RA, CCL2, CXCL12), cell- and matrix-modulating (SCF, M-CSF, OPG) and angiogenetic factors (TIMP2, VEGF) were detected following biocompatible treatment with CPP. Osteogenic stimulus and CPP treatment resulted in upregulation of CCL2 (non-osteogenic culture conditions) and IL1-RA (osteogenic culture conditions) at the early time point (day 4). CCL2 and IL-1RA are known to play a major role in MSC-based tissue repair/wound healing by inducing immunosuppressive effects [44,45]. Secretion of anti-inflammatory CXCL12 was found to be downregulated at day 8 after exposure to CPP at both nonosteogenic and osteogenic culture conditions. The chemokine CXCL12 (SDF1) is known to activate immunosuppressive macrophages and is involved in tissue repair and immune maintenance [46]. Another interesting finding of the presented in vitro study is the regulation of M-CSF and OPG secretion by BM-MSCs after osteogenic stimulus and CPP treatment. M-CSF and OPG play a crucial role in the differentiation of macrophages and in the regulation of osteoclast activity, respectively and thus in the formation and functionality of bone resorbing osteoclasts [47,48]. The downregulation of M-CSF secretion after CPP exposure at osteogenic conditions suggests a paracrine effect leading to diminished macrophage differentiation and consequently to reduced bone resorption. In contrast, the diminished secretion of OPG after CPP exposure at day 8 of osteogenic differentiation suggest a paracrine effect leading to enhanced bone resorption. Advanced models, e.g., osteoblast and osteoclast cocultures are needed to investigate the effects of CPP at biocompatible dose on osteoclast formation and activity [49]. Sufficient osteointegration of arthroplasty implants and vital bone healing is associated with sufficient angiogenesis in the surrounding tissue [50]. The data of our study suggest that CPP exposure leads to a proangiogenetic secretion profile of hBM-MSCs, by a significant decrease of angiogenesis inhibiting matrix-metalloproteinase TIMP2 at day 4 and day 8 of osteogenic differentiation and a significant increase of the proangiogenetic growth factor VEGF at day 4 of osteogenic differentiation [51,52].

Taken together, the presented study is a first step towards preclinical testing of CPP in the context of musculoskeletal regeneration. The effects of CPP on primary cells of the musculoskeletal system remains relevant to explore due to the need for local/intraoperative therapeutic approaches of bone pathologies associated to inflammation. The in vitro data shown here were obtained with primary cells from a relevant patient clientele. The strength of our work is that we were able to demonstrate heterogeneity due to donor-individual differences by the chosen experimental approach. Notably, CPP should not be considered a miracle cure. The properties and the respective potential for treatment are linked many factors such as gas mixture and dosimetry. Therefore, it is crucial to consider the influence of these factors in the context of preclinical in vitro studies.

## 4. Material and Methods

### 4.1. Patient Recruitment and Sample Harvest

Metaphyseal BM samples were harvested from the femoral marrow cavity during primary implantations of hip arthroplasty implants. Overall, 10 patients with indication for joint replacement due to osteoarthritis of the hip joint were enrolled. Cells from 5 female and 3 male patients with a median age of 76.1 ± 9.0 years were used for the experimental workflow of this study. Demographic data as well as primary and secondary medical data are depicted in Table 1. Ethics approval (BB 160/20) was obtained from the local independent ethics committee (IEC) of the University Medicine Greifswald according to the World Medical Association Declaration of Helsinki. All donors gave written informed consent.

### 4.2. Cell Isolation, Cultivation and Characterization

Human bone-marrow-derived mononuclear cells (hBM-MNCs) were isolated via density gradient centrifugation as described previously [53]. To isolate hBM-MSCs via plastic adherence, hBM-MNCs were plated on tissue culture flasks at a cell density of 5 × 10^5^/cm^2^ and cultured under standard cell culture conditions (37 °C, 5% CO_2_) in expansion medium containing low glucose DMEM (PAN Biotech, Aidenbach, Germany) supplemented with 10% human platelet lysate (HPL, PAN Biotech, Aidenbach, Germany), 100 U/mL penicillin (Gibco, Waltham, Massachusetts, USA), 100 μg/mL streptomycin (Gibco) and 2 mM L-alanyl-L-glutamine (GlutaMAX, Gibco, Waltham, Massachusetts, USA). Nonadhering cells were removed after 48–72 h. Media change was conducted twice a week, and cells were trypsinized at 80% confluence. After reaching cell culture passage two, hBM-MSCs were cryopreserved in low glucose DMEM supplemented with 12.5% human serum albumin (Biotest Pharma GmbH, Dreieich, Germany) and 10% DMSO (AppliChem, Darmstadt, Germany). MSC characterization was performed by cell surface marker analysis. To this end, MSCs were thawed and cultured in expansion medium until cell culture passage number three before detachment with accutase (PAN Biotech, Aidenbach, Germany) to obtain single-cell suspensions for subsequent flow cytometry analysis. Cells were washed twice in PBS (Bio&Sell GmbH, Feucht, Germany) and incubated for 15 min at RT in the dark with fluorescently labeled monoclonal antibodies (all BioLegend, San Diego, CA, USA) targeting CD73 (brilliant violet 421), CD90 (phycoerythrin), and CD105 (PerCP cyanine 5.5.) to label MSC as well as CD19, CD14, CD19, CD34, CD45, and HLA-DR (all APC cyanine 7) to label non-MSCs. After washing, cells were suspended in FACS buffer and analyzed using flow cytometry (CytoFLEX LX, Beckman-Coulter, Brea, CA, USA).

### 4.3. In Vitro Exposure to Cold Physical Plasma

In this study, the atmospheric pressure argon plasma jet kINPen (neoplas Med GmbH, Greifswald, Germany) was utilized. Details on its applications and safety profiles as well as construction and technical design were summarized previously [8,54]. Argon gas (Air Liquide, two standard liters per minute) was excited by a high-frequency electrode inside the jet to generate ROS/RNS after expulsion to ambient air containing. For in vitro treatment, the jet was attached to a *xyz*-motorized precision controller stage (CNC), hoovering the gas plasma above the center of each well for the set time. Evaporation of liquid was compensated by adding predetermined amounts of double-distilled H_2_O to each well after treatment.

### 4.4. Quantification of Reactive Oxygen and Nitrogen Species

To quantify hydrogen peroxide (H_2_O_2_), one of the final products of the short-lived reactive species chemistry generated by the gas plasma, the amplex ultra red assay (ThermoFisher, Waltham, Massachusetts, USA), was used according to the manufacturer’s instructions. For the detection of two other stable molecules, nitrite (NO_2_^-^) and nitrate (NO_3_^-^), the Griess assay (ThermoFisher, Waltham, Massachusetts, USA) was performed according to the manufacturer’s instructions. As liquid carriers, PBS and cell culture media were used. Quantification was performed by comparing to standards with known H_2_O_2_, NO_2_^-^ and NO_3_^-^ concentrations. The ROS/RNS quantification assays were performed under equal CCP treatment regimes as for treatment of cells, i.e., 200 µL in 48-well plates.

### 4.5. Cell Viability and Proliferation

hBM-MSCs from cell culture passage two were thawed and cultured in expansion medium to reach 80% confluence. After trypsinization, 1.8 × 10^3^ cells were seeded on 48-well tissue culture plates (30% confluence). After obtaining sufficient cell attachment by culturing for 24 h in 200 µL expansion medium, the cells were washed with PBS and frozen at −80 °C for later quantification of the initial DNA content (CyQuant assay, ThermoFisher, Waltham, Massachusetts, USA) according to the assay manual, i.e., for calculation of cell population doublings. In all other plates, the medium was exchanged with 200 µL fresh expansion medium and the cells were treated for either 30 s (t1), 60 s (t2), 120 s (t3), or with nonignited working gas (control). The cells were cultivated for 4 days prior to quantification of cellular metabolic activity as a marker for cell viability via a resazurin-based assay (PrestoBlue, Invitrogen, Waltham, Massachusetts, USA) according to the assay manual. After media exchange, independent culture plates were further cultivated to quantify cell viability after 8 days of culture. At both time points, the respective well plates were washed with PBS after cell viability quantification and frozen overnight at −80 °C for subsequent DNA determination via CyQuant assay (ThermoFisher, Waltham, Massachusetts, USA) according to the manufacturer’s instruction, to evaluate the influence of CPP on proliferation rates. The fluorescence intensities of the CyQuant assay of days 4 and 8 were compared to the initial values (24 h after seeding) to calculate the number of population doublings.

### 4.6. High Content Fluorescence Imaging

Patient-derived hBM-MSCs from cell culture passage two were thawed and cultured in expansion medium to reach 80% confluence. After trypsinization, 9.0 × 10^3^ cells were seeded in 48-well tissue culture plates in 200 µL expansion medium per well. After 24 h of incubation, the medium was exchanged with 200 µL of fresh expansion medium, and the cells were treated with CPP for 30 s (t1), 60 s (t2), or 120 s (t3). After another 24 h of incubation, cells were stained with MitoSpy green (BioLegend, San Diego, CA, USA), followed by fixation with 4% paraformaldehyde, washing, and staining with Flash Phalloidin Red (BioLegend, San Diego, CA, USA) and 4′,6-Diamidin-2-phenylindol (DAPI, final concentration 1 µM; ThermoFisher, Waltham, Massachusetts, USA) in permeabilization wash buffer (BioLegend, San Diego, CA, USA). Finally, cells were washed, and PBS was added. Microscopy was performed using a high content imaging device (Operetta CLS, PerkinElmer, Waltham, Massachusetts, USA) using 5× (NA = 0.16) and 63× water immersion (NA = 1.16) objectives in epifluorescence and spinning-disc confocal mode, respectively. Bandpass filter centers were λ_ex_ 365 nm and λ_em_ 465 nm for DAPI, λ_ex_ 475 nm and λ_em_ 525 nm for MitoSpy, and λ_ex_ 550 nm and λ_em_ 610 nm for Flash phalloidin. Several z-stacks were acquired for each field of view to calculate maximal intensity projections, and several fields of view were acquired per well. For quantitative image analysis and algorithm-based and nonmanual, unsupervised object segmentation, Harmony 4.9 software (PerkinElmer) was used. After image acquisition, sliding-parabola functions were utilized to enhance signal-to-noise ratios and by this reducing background. Cell regions were detected using a threshold of pseudofluorescence channels (combined from all fluorescence channels) in relation to the background of every single image separately. Prior to this, the problem of uneven lighting of the wells was digitally corrected using flatfield correction. Mitochondria were detected as fluorescent spots inside this cell region with distinct discrimination values, as their size, relative intensity, and contrast of the spots to the background and each other (splitting). For texture analysis, a texture SER image method was used and speckle and ridge filters were applied using Kernel normalization to 1-pixel units, followed by quantitative analysis of object features. The settings of the texture filters were identical across all fields of view analyzed, as the curvature of the sliding parabola was kept the same. 

### 4.7. In Vitro Stimulation of Osteogenic Differentiation

Expansion of hBM-MSCs for the osteogenic differentiation assay was performed with media containing 10% fetal bovine serum (FBS, Sigma Aldrich, St. Louis, MO, USA). hBM-MSCs from cell culture passage two were thawed and expanded to 80% confluence. After trypsinization, 6.0 × 10^3^ were seeded on 48-well tissue culture plates (100% confluence). After obtaining sufficient cell attachment by culturing for 24 h in 200 µL expansion medium, the cells were stimulated for osteogenic differentiation with medium containing 10% FBS, 50 µM L-Ascorbic acid 2-phosphate sesquimagnesium salt, 10 mM ß-Glycerolphosphate disodium salt, 100 nM dexamethasone (all Sigma Aldrich, St. Louis, MO, USA), 100 U/mL penicillin, 100 μg/mL streptomycin, and 2 mM L-alanyl-L-glutamine (GlutaMAX). An equal number of wells supplemented with expansion medium (10% FBS) served as negative controls for osteogenic differentiation. Directly after media change, the cells were treated for 30 s (t_1_) or with non-ignited working gas (control) for the first time. The same CPP treatment regime was repeated on culture day 4. Osteogenic differentiation of each experimental condition was conducted in triplicates.

### 4.8. Quantification of the Osteogenic Differentiation Potential

Cellular ALP activity was quantified at days 4 and 8 of osteogenic differentiation by colorimetric quantification of 4-nitrophenolate accumulation after 10 min and incubation at 37 °C with para-Nitrophenylphosphate (Sigma Aldrich, St. Louis, MO, USA) as previously described [54,55]. Human procollagen type I N-terminal propeptide (P1NP) concentrations in the cell culture supernatant were quantified by ELISA assay (Elabscience, Wuhan, China) according to the kit manual at days 4 and 8 of osteogenic differentiation. Osteogenic matrix mineralization was quantified at day 11 of osteogenic differentiation by staining of calcium deposition in osteogenic matrix with Alizarin Red S (Sigma Aldrich, St. Louis, MO, USA) solution and subsequent colorimetric quantification of the cetylpyridinium chloride (Sigma Aldrich, St. Louis, MO, USA) dissolved calcium matrix as previously described [56].

### 4.9. Soluble Mediator Multiplex Analysis

A customized panel (LEGENDplex Custom Human 12plex Panel, BioLegend, San Diego, CA, USA) was designed to analyze relevant hBM-MSC-secreted cytokines and growth factors involved in immune cell recruitment, anti-inflammatory pathways, matrix remodeling and bone metabolism. This included CCL2 (MCP-1), CXCL12 (SDF1), CXCL8 (IL-8), HGF, IL-1RA, IL-6, M-CSF, OPG, SCF, TGF-β (Free Active), TIMP-2 and VEGF. Media supernatant was collected at days 4 and 8 of osteogenic differentiation and stored at −80 °C until multiplex analysis. Supernatant from two wells of each treatment condition was pooled for cytokine detection. The analysis was performed with media supernatant from six donors according to the manufacturer’s instructions.

### 4.10. Data Analysis, Presentation, and Statistics

No samples or technical replicates were excluded from the statistical analyses. Sample size was not predetermined by statistical methods. Randomization was not applied and the investigators were not blinded to group allocation during the experiments. GraphPad Prism 8.4.3 was used for exploratory statistical analyses and descriptive data plotting. Flow cytometry data analysis was performed using Kaluza software 2.1 (Beckman-Coulter, Brea, CA, USA). An algorithm-based and nonmanual object segmentation, Harmony 4.9 software (PerkinElmer, Waltham, Massachusetts, USA) was used for quantitative image analysis of images obtained by high content fluorescence imaging. Images were flatfield-corrected, and sliding-parabola functions were utilized to enhance signal-to-noise ratios. For texture analysis, speckle and ridge filters were applied, followed by quantitative analysis of object features. For data extraction, all image analysis steps were the same for all wells and conditions analyzed across the 3000 images included in this data set. Detailed information about error bars, sample sizes per group, and experiment-specific statistical analysis are included in figure legends.

## 5. Conclusions

The obtained data demonstrate that the treatment of hBM-MSCs at biocompatible doses of clinically approved kINPen CPP (1) did not promote in vitro osteogenic differentiation, (2) did not impair hBM-MSCs functionality, (3) did not induce excessive release of pro-inflammatory cytokines and chemokines and (4) led to modest but significant augmentation of anti-inflammatory and proangiogenic cytokines and growth factors. By using hBM-MSCs from a clinically relevant orthopedic patient cohort, the data of this study contributes to the further preclinical evaluation of CPP. This is crucial for therapeutic improvement in musculoskeletal regeneration accompanied by keeping patient’s safety at the highest possible level. We are convinced that clinical CPP-treatment has the potential to become a powerful therapeutic approach in musculoskeletal medicine. Furthermore, testing of CPP in in vivo models or in multicellular in vitro models is indicated to understand the comprehensive influence of CPP on the complex human bone homeostasis and to further preclinically evaluate CPP safety and efficacy.

## Figures and Tables

**Table 1:** Baseline patient and sample data.

Patient	Age	Sex	Indication for Surgery	Surgical Treatment	Long-Term Medication	Comorbidities	BM Weight (g)	Number of BM-MNCs Isolated (×10^6^)
donor 1	75.7	w	hip osteoarthritis	primary THA	pantoprazole, lisinopril, bisoprolol, allopurinol, prednisolone	polymyalgia rheumatica, art. hypertension, anxiety disorder	10.56	144
donor 2	81.5	w	hip osteoarthritis	primary THA	amlodipine, olmesartan	art. hypertension	6.56	62
donor 3	79.5	w	hip osteoarthritis	primary THA	acetylsalicylic acid, bisoprolol, ramipril, torsemide, cholecalciferol	art. hypertension, osteoporosis, chronic kidney disease	4.12	149
donor 4	53.2	w	developmental dysplasia of the hip	primary THA	-	-	6.31	210
donor 5	68.1	m	hip osteoarthritis	primary THA	allopurinol, pantoprazole	dyslipidemia	7.53	230
donor 6	77.1	m	hip osteoarthritis	primary THA	ibuprofen, acetylsalicylic acid, simvastatin, candesartan, hydrochlorothiazide	B-cell-lymphoma, art. hypertension, chronic back pain, stroke, lumbar disc disease	6.33	279
donor 7	76.4	w	hip osteoarthritis	primary THA	acetylsalicylic acid, carvedilol, amlodipine, trimipramine, ezetimibe/simvastatin, candesartan	art. hypertension, dyslipidemia, diabetes mellitus, depression, aortic valve insufficiency	2.23	104
donor 8	75.3	m	hip osteoarthritis	primary THA	irbesartan, hydrochlorothiazide, carvedilol	art. hypertension, obesity	8.55	203

## Data Availability

Not applicable.

## Supplementary Materials

The following supporting information can be downloaded at: www.mdpi.com/article/10.3390/ijms23042038/s1.
