# Supplementary material for: Biocompatible Gas Plasma Treatment Affects Secretion Profiles but Not Osteogenic Differentiation in Patient-Derived Mesenchymal Stromal Cells
Source: Int J Mol Sci. 2022 Feb 12;23(4):2038. doi: 10.3390/ijms23042038 (PMC8879607; doi:10.3390/ijms23042038)
Supplement: Supplementary file 1 [file ijms-23-02038-s001.zip › ijms-1585266-supplementary.pdf]

Biocompatible Gas Plasma Treatment Affects Secretion Profiles but Not Osteogenic Differentiation in Patient-Derived Mesenchymal Stromal Cells

Maximilian Fischer [1], Janosch Schoon [1,*], Eric Freund [2,3], Lea Miebach [2,3], Klaus-Dieter Weltmann [2], Sander Bekeschus [2,*,†] and Georgi I. Wassilew [1,†]

[1] Center for Orthopaedics, Trauma Surgery and Rehabilitation Medicine, University Medicine Greifswald, 17475 Greifswald, Germany; maximilian.fischer@med.uni-greifswald.de (M.F.); georgi.wassilew@med.uni-greifswald.de (G.I.W.)
[2] ZIK *plasmatis*, Leibniz Institute for Plasma Science and Technology (INP), Felix-Hausdorff-Str. 2, 17489 Greifswald, Germany; eric.freund@inp-greifswald.de (E.F.); lea.miebach@inp-greifswald.de (L.M.); weltmann@inp-greifswald.de (K.-D.W.)
[3] Department of General, Visceral, Thorax, and Vascular Surgery, Greifswald University Medical Center, 17475 Greifswald, Germany
* Correspondence: janosch.schoon@med.uni-greifswald.de (J.S.); sander.bekeschus@inp-greifswald.de (S.B.)
† These authors contributed equally as senior authors.

Supplementary Material

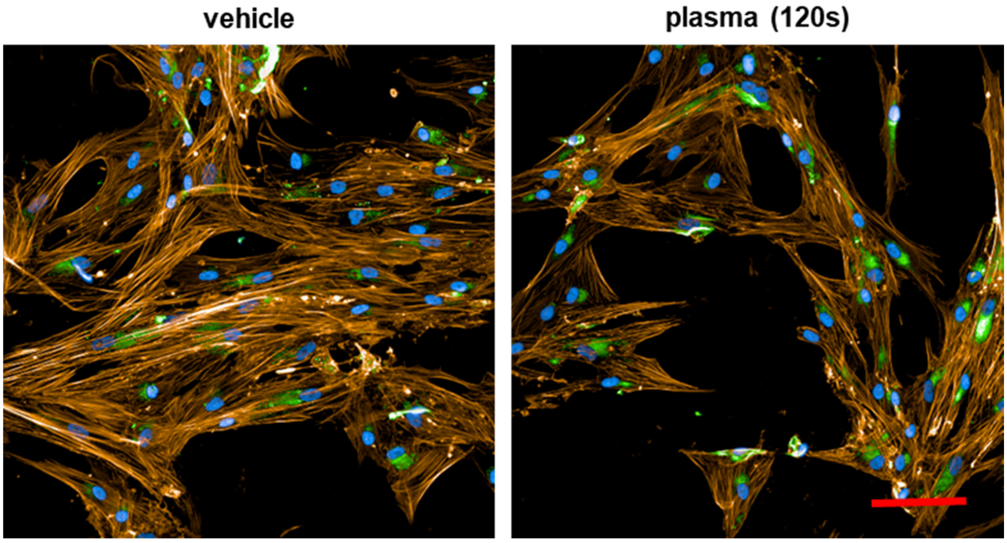

Figure S1. Representative 3-channel fluorescence image of control (vehicle) and plasma-treated (120 s) cells. Scale bar: 100 μm.
